# CCNA2 and CCNB3 as Early Potential Molecular Candidates of Oocyte Maturation in Cumulus-Oophorous Complex Cells from Follicular Fluid

**DOI:** 10.3390/diagnostics15202658

**Published:** 2025-10-21

**Authors:** Nergis Özlem Kılıç, Çağrı Öner, Duygu Kütük, Belgin Selam, İbrahim Orçun Olcay, Ertuğrul Çolak

**Affiliations:** 1 Department of Histology and Embryology, Medical Faculty, Maltepe University, 34844 Istanbul, Turkey; duygukutukk@gmail.com; 2 Department of Medical Biology, Medical Faculty, Kırklareli University, 39100 Kırklareli, Turkey; cagrioner@klu.edu.tr; 3 Department of Obstetrics and Gynecology, School of Medicine, Acıbadem Mehmet Ali Aydınlar University, 34752 Istanbul, Turkey; 4 IVF Laboratory, Bahçeci Umut Assisted Reproduction Center, 34662 Istanbul, Turkey; oolcay@bahceci.com; 5 Department of Biostatistics, Medical Faculty, Eskişehir Osmangazi University, 26040 Eskişehir, Turkey; ecolak@ogu.edu.tr

**Keywords:** embryology, epigenetic imprinting, microRNA, oocyte pick up (opu), oogenesis, cell cycle proteins

## Abstract

**Background/Objectives**: Oocyte maturation is a process involving both nuclear and cytoplasmic development regulated by epigenetic changes in gene expression. Cyclin-B3 (CCNB3) and cyclin-A2 (CCNA2) genes are thought to be involved in oocyte maturation; however, the expression profiles and key function in Metaphase-I (MI) and Metaphase-II (MII) phases have yet to be fully elucidated. Small non-coding RNA sequences are involved in epigenetic regulation of specific transcriptional targets, whereas microRNAs (miRNAs) participate in the post-transcriptional and translational repression of target genes. This study examined the expression levels of CCNB3, CCNA2, and their associated miRNAs (miR-17, miR-106b, miR-190a, miR-1275) in cumulus oophorous complex (COC) cells derived from MI and MII oocytes of NOR and DOR IVF cases, with particular emphasis on elucidating their functions during the transition from MI to MII stage. **Methods**: Follicular fluid containing cumulus–oocyte complex (COC) cells obtained from oocytes of 120 cases in each group NOR MI (*n* = 30), NOR MII (*n* = 30), DOR MI (*n* = 30), and DOR MII (*n* = 30) who were admitted to the Istanbul Bahçeci Health Group Assisted Reproductive Treatment Center. Following total RNA isolation from COC cells, the gene and protein expression levels of CCNB3 and CCNA2, along with the expression of miR-17, miR-106b, miR-190a, and miR-1275, were assessed using (qPCR-based assay) and immunohistochemistry (IHC). To investigate the functional roles of COC cell populations, morphological analysis was performed using H&E staining. Additionally, metadata of the cases, including age, number of oocytes, fertilization, and embryonic development rates, were evaluated. **Results**: The expressions of miR-17 and miR-1275 were significantly elevated in both NOR MI and DOR MI groups compared to their respective NOR MII and DOR MII groups (*p* < 0.05). Additionally, miR-106b levels were higher in the NOR MII group relative to NOR MI (*p* < 0.05), while an increase was also observed in DOR MI compared to DOR MII (*p* < 0.05). No difference was observed in miR-190a expression between the NOR and DOR (*p* > 0.05). Based on the results of H and E staining, the NOR MI, NOR MII, DOR MI, and DOR MII groups exhibited distinct variations in cellular morphology, nuclear characteristics, cytoplasmic volume, and cell density. **Conclusions**: CCNB3 is predicted to be a potential candidate for determining MI between the NOR and DOR cases. On the other hand, only for the NOR MII cases could CCNA2 provide evidence of oocyte maturation. Moreover, we determined the relationship between related genes and miRNAs which target CCNA2 and CCNB3. Genetic and protein expression analysis across diverse molecular pathways and miRNAs yielded comprehensive preliminary data regarding the developmental stages of oocytes at the MI and MII phases, and their fertilization potential following maturation shows potential and warrants prospective validation with clinical performance evaluation.

## 1. Introduction

Infertility is defined as the inability to conceive after approximately 12 months of unprotected and regular sexual intercourse, and approximately 15% of couples require medical assistance due to infertility [1]. Causes of female infertility are divided into subgroups such as abnormalities related to oocyte production, cervical and tubal factor infertility, uterine factors, endometriosis, immunological factors, and unexplained infertility [2]. In Vitro Fertilization (IVF) is a procedure involving the artificial fertilization of oocytes and spermatozoa outside the uterus. [3]. The IVF procedure comprises several fundamental stages: ovarian stimulation and monitoring, follicle retrieval (oocyte pick up, or OPU), oocyte fertilization [4], embryo development and, ultimately, the transfer of the embryo to the uterus [5].

Oocyte maturation is a process that refers to the release of meiotic arrest, allowing oocytes to progress from prophase in meiosis I to metaphase in meiosis II. This process includes both nuclear and cytoplasmic maturation. While nuclear maturation involves chromosomal segregation, cytoplasmic maturation is important in order for the oocyte to become ready for fertilization [6]. The metaphase II oocyte, which is released during ovulation, can complete meiosis II upon fertilization [7]. The quality of the follicular microenvironment surrounding the oocytes and the continuity of cell communication are very important for the proper and healthy completion of oocyte development [8]. Fertilization and consequent embryo development are dependent on complex molecular processes that begin with the development of oocyte competence through maturation. The acquisition of a mature oocyte is associated with transcriptomic transitions characterized by specific transcriptional conditions such as folliculogenesis and oogenesis, which are multi-stage developmental mechanisms which lay the groundwork for early embryonic development [9]. The oocyte transcriptome is a dynamic process that requires control and is regulated by multiple pathways that are activated and repressed in oocytes at different follicular stages during oocyte growth and maturation [10]. While the primordial stage reflects the strong communication between the oocyte and surrounding cells, antral follicles show down-regulation of cytoplasmic translation [11]. The expression levels of genes associated with oocyte development in cumulus oophorous complex (COC) cells which surround the oocyte directly have been found to correlate with oocyte maturation [12], embryo development capacity [13], and live birth rates [14].

Non-coding RNAs are functional RNA molecules that are not translated into proteins. They are divided into two main groups: long non-coding RNAs and short non-coding RNAs. The short non-coding RNA members are short interfering RNA (siRNA), microRNA (miRNA) and PIWI-interacting RNA (piRNA) and are closely associated with transcription, translation and post-transcriptional inhibition mechanisms [15]. miRNAs are short non-coding RNA molecules that function as gene regulators in many biological systems, including the oocyte and embryo development. While siRNAs epigenetically regulate the expression of their target genes, miRNAs are involved in post-transcriptional and translational repression of mRNA from target genes [16]. The resumption of meiosis in the oocyte maturation process and the control of the bidirectional exchange of substances by the cumulus cells on the oocyte suggest that the continuation of cell metabolism is physiologically dependent on the use of resources outside the oocyte. It has been suggested that this mechanism is necessary for transcriptional silencing and may be linked to the transcription of stored mRNA or post-transcriptional modification events [17]. Studies on miRNAs have revealed that they have important functions in the oocyte maturation process as main regulators of cell differentiation processes. Studies in mice [18] and pigs [19] have reported dynamic changes in the expression levels of different miRNA profiles associated with follicle development and maturation. Some of the miRNAs found in granulosa and COC cells are miR-17, -27, -92a, -106b and -145, and they improve the chromatin configuration rate in the maturation processes of oocytes. miR-17 is especially effective at improving the developmental ability of oocytes until the blastocyst stage is reached [20]. To investigate the roles of miRNAs in oocyte maturation in more depth, miRNA sequencing was used to characterize miRNA populations present in bovine germinal vesicle (GV) oocytes, metaphase II (MII) oocytes, and possible zygote pools, indicating that each stage contains a defined miRNA population. Bos taurus (bta)-miR-155, bta-miR-222, bta-miR-21, bta-let-7d, bta-let-7i, and bta-miR-190a were identified as statistically significantly expressed miRNAs, while others showed progressive changes between stages [21]. In a study conducted by Zhang et al. in rats with premature ovarian failure (POF) miRNA-190a-5p showed a significant differential expression profile in POF cases because it continuously activate primordial follicles in rats by targeting the expression of the PH site and leucine-rich repeat protein phosphatase 1 (PHLPP1) gene in both protein kinase-B transcription factor protein O3 (AKT-FOXO3a) and AKT-luteinizing Hormone/Luteinizing Hormone Receptor (AKT-LH/LHR) pathways. The existence of increased AKT kinase signaling pathways was observed through chemical environment-induced POF models, and resulted in primordial follicular hyperactivation. It has even been demonstrated that phosphorylated AKT can trigger the phosphorylation of several downstream proteins (FOXO3a, BCL-2,) following direct or indirect activation of AKT, thereby promoting the growth and proliferation of follicular cells. Furthermore, it has been documented that upstream molecules’ activation of AKT hyperphosphorylated FOXO3a transports it out of the nucleus and promotes the beginning of primordial follicles. Thus, it has been concluded that miRNA-190a-5p could provide potential evidence of early recognition of POF cases [22].

A study involving miR-1275 in porcine oocytes concluded that miR-1275 is expressed in follicular oocytes and promotes early apoptosis in granulosa cells (pGCs). This function suppressed estradiol (E2) synthesis by attenuating liver receptor homolog-1 (LRH-1) expression and preventing the interaction of LRH-1 protein with cytochrome P450 family 19A (1CYP19A1) promoter. As a result, miR-1275 initiates follicular atresia in pig ovaries by promoting apoptosis in pGCs [23].

Cyclins (CCNs) and cyclin-dependent kinases (cdks) are enzymes that play an important role in cell cycle regulation. Cdks require a subunit called CCN for their enzymatic activity and CCNs bind to CDKs and lead to their enzymatic activity. This interaction is critical for cell division and progression. They regulate the progression of the cell cycle through various mechanisms such as CCN synthesis and degradation, posttranslational modification in the growth phase (G1), the DNA synthesis phase (S), the division preparation phase (G2), and the mitosis and cytokinesis phases (M). The CCN/cdk complex represses the retinoblastoma (Rb) protein through phosphorylation and promotes entry into the S phase of interphase and in the S phase CCNA2/cdk2 complex regulates entry into the G2 phase of the cell cycle interphase [24]. In the late G2 phase, CCNA2 activates cdk1 to facilitate the initiation of M phase and the CCNB/cdk1 complex guides the cell to the G2-M transition. The highest level of activity of the CCNB/cdk1 complex is observed in the M phase of mitosis and only cdk1, CCNA2 and CCNB1 are required for mitotic cell cycle progression [25] and a study indicated that cdk1, CCNA2 and CCNB1 factors are especially essential for embryonic development [26].

CCNA2 was first identified in studies in oyster embryos as one of a group of proteins that are cyclically synthesized and degraded at the beginning of the M phase of the cell cycle [27]. The CCNA2 gene is initially expressed in the S phase of the interphase during DNA replication and gradually decreases until it completes the degradation process in the prometaphase of the M phase [28]. In a study by Swenson et al. (1986), it was shown that injecting cyclin-A mRNA into Xenopus oocytes was sufficient to cause oocytes in the G2 phase to progress to M phase [29]. The oocytes in which the CCNA2 gene was inhibited demonstrated anomalies in the formation of meiosis-II spindle formation; in other words, there was an increase in the incidence of merotelic kinetochore–microtubule attachment with chromosome formation after MII phase while MI spindles could form [30]. Furthermore, it has been suggested that without CCNA2 gene expression, regulating and repairing defects in chromosome–kinetochore attachment may be difficult [31]. Although the CCNB3 gene is classified as a B-type cyclin, it has features of both A and B-type cyclins. In contrast to CCNB1 and CCNB2 gene expression, the CCNB3 gene is specifically expressed in mammalian germ cells [32]. A study in Drosophila reported that the CCNB3 gene promotes the metaphase–anaphase transition in early embryonic divisions and required for meiosis, especially in females [33]. The CCNB3 gene inhibited mice, which showed morphologically normal mature ovarian follicle development but were unable to transition from the metaphase I to anaphase I stage. Furthermore, it has been reported that CCNB3 gene-inhibited oocytes were unable to form polar bodies (PB), however could only form oocytes without PB at metaphase I stage [34]. A study by Karasu et al. (2019) [35] demonstrated that in CCNB3-null oocytes, the CCNB1 gene and securin were not degraded, resulting in sustained MPF activity and a prolonged metaphase-I (MI) phase. Consequently, this delay prevented progression into the anaphase stage [35,36].

According to the literature, the role of cyclins in oocytes during mitosis and meiosis has been examined through genetic studies, which have demonstrated that both cyclins and cyclin-dependent kinases (CDKs) are integral to cell cycle regulation. While CCNB3 and CCNA2 gene expressions are recognized as important during the MI and MII phases of oocyte maturation, further research is needed to elucidate their specific expression patterns and the epigenetic mechanisms that regulate them in relation to oocyte maturation and fertilization rates.

## 2. Materials and Methods

Women who were referred to Bahçeci Health Group Umut IVF Center between 22 April 2024–22 August 2025 were included in the study (Ethics Committee No: 2024/06-08; written informed consent was obtained from all study participants). In this study, we collected and researched COC samples in the follicular aspiration fluids of NOR MI, NOR MII, DOR MI, and DOR MII cases. A total of 120 COC samples were collected from each of the two groups as [normoresponder (NOR; *n* = 30); intermediate grouped NOR metaphase-I (MI; *n* = 30) and metaphase-II (MII; *n* = 30); diminished ovarian reserve (DOR; *n* = 30); intermediate grouped DOR metaphase-I (MI; *n* = 30) and metaphase-II (MII; *n* = 30)]. Inclusion criteria for the NOR (control group) cases AFC 7-12, and anti-Mullerian Hormone (AMH) levels 1.0–3.5 ng/mL. DOR cases were required to meet the following criteria: AFC < 7, AMH levels > 1 ng/mL. In each group, patients provide a minimum of two oocytes, as cumulus–oocyte complex (COC) cells were to be collected based on MI and MII parameters in both the NOR and DOR groups. Cases from either group who had only one oocyte collected were excluded from the study.

### 2.1. Sample Size and Power

For sample-size justification, we focused on the clinically most relevant contrast in our design: the MI vs. MII difference in CCNA2 ΔCt within the NOR stratum. In the results, the NOR group showed MI = 0.354 ± 2.787 vs. MII = 3.962 ± 3.584 (mean ± SD; per-phase *n* = 30; total *N* = 120 across NOR/DOR × MI/MII). Using the observed SDs as variance estimates for sensitivity (not “observed/post hoc” power), the pooled SD was 3.210. The observed mean difference was 3.608 ΔCt, corresponding to Cohen’s d = 1.124 (Hedges’ g = 1.109; 95% CI for d: 0.58–1.67). The 95% CI for the mean difference was 1.95–5.27 ΔCt. With *n* = 30 per group, the minimal detectable difference (MDE) at 80% power and α = 0.05 (two-sided) is about 2.32 ΔCt, computed as Δ_min ≈ (z_{1 − α/2} + z_{1 − β}) · s_*p* · √(2/*n*). Because the observed difference (3.61 ΔCt) materially exceeds the MDE (2.32 ΔCt), the available sample size provides adequate sensitivity for the primary contrast. To align with the reviewer’s request on fold changes, a ΔCt difference in Δ translates to a relative fold change of 2^{|Δ|}; hence MDE = 2.32 ΔCt ≈ 5-fold, while the observed difference 3.61 ΔCt ≈ 12.2-fold. We report effect sizes with 95% CIs and MDE rather than “observed power,” which is mathematically redundant with the *p*-value. Assumptions for the sensitivity calculation were equal variances and independent observations; when distributional assumptions are not strictly met, this calculation is conservative.

### 2.2. Isolation of Cumulus Oophorus Cells (COCs) from Follicular Fluid of NOR and DOR Cases

Cumulus oophorous complex (COC) cells were isolated from the follicular fluid (FF) of NOR and DOR cases’ MI and MII phases during the oocyte pickup (OPU) procedure. The denuding process was used to remove cumulus cells (CCs) with corona radiata cells which form the structure of cumulus oophorous complex (COC) cells from each individual. Using sensitive glass pipettes, COC cells were mechanically separated from oocytes under stereomicroscopy. In the TCM199 medium containing 20% fetal calf serum (FCS) and 80 IU hyaluronidase/mL, COC cells were carefully pipetted up and down. Polar body (PB) visualization under a stereomicroscope (Nikon SMZ-1500, Nikon, Berlin, Germany) was used to investigate the oocyte meiotic stage following the total removal of COC cells from the oocytes. The collected cases’ COC cells were placed in centrifuge tubes in their FF separately, according to MI and MII oocytes. Then, FF with COC cells was centrifuged at +4 °C and 2800 rpm for 20 min. The supernatant was discharged, and pellets which included COC cells were centrifuged at 1850 rpm at room temperature for 5 min in phosphate-buffered saline (PBS).

### 2.3. Total RNA Isolation from Cumulus Oophorus Cells (COC) and Determination of Gene Expression and miRNA Expression by Quantitative Real-Time Polymerase Chain Reaction (qPCR-Based Assay)

Total RNA isolation performed according to NGE024 NucleoGene QuickEX Total RNA Isolation Kit protocol and cDNA was created by reverse transcription of total RNAs (Nucleogene, Istanbul, Turkey). The cDNA reverse transcription was performed at 25 °C for 5 min, 50 °C for 30 min, and 85 °C for 5 min. SYBR Green primer sets used for the amplification of Cyclin-B3 (CCNB3), Cyclin-A2 (CCNA2), mikro-RNA (miR-17, miR106b, miR1275 and miR-190a) and Glyseraldehide-3-phosphate dehydrogenase (GAPDH) were designed and supplied by Bmlabosis (Ankara, Türkiye). The isolated samples were measured for RNA on a Microplate Reader (Spectrostar Nano, BMG-Labtech, Ortenberg, Germany) and stored at −80 °C (Haier Biomedical, Qingdao, China) for further processing. The primer sequences are shown in Table 1. Glyceraldehyde-3-phosphate dehydrogenase (GAPDH) was used as an internal control to calculate of ΔCT values (Table 1, Supplementary Materials S1).

### 2.4. Determination of CCNA2 and CCNB3 Protein Expression by Immunohistochemistry (IHC)

CCNA2 and CCNB3 protein expression was investigated according to the Envision IHC Staining kit (Dako Omnis, Agilent, Singapore) protocol. NOR and DOR cases’ COC cells isolated from FF were prepared as smears on a polylysine-coated slide (Objekttrager, Adhesive-Silane, Heidelberg, Germany) and dried at room temperature. After fixation in 37% formaldehyde solution for 15 min, CCNB3 and CCNA2 primary antibodies (DF10120 and AF0142 Affinity Biotech) were diluted in 0.1% (*w*/*v*) bovine serum albumin (BSA) and incubated with primary antibodies at +4 °C overnight. The next day, the samples were washed with (PBS) Phosphate-Buffered Saline (PBS) (Sigma Aldrich, Taufkirchen, Germany) and incubated with EnVision FLEX Peroxidase-Blocking Reagent (Dako Omnis, Singapore) for 3 min at room temperature to block non-specific binding and washed with PBS. As a secondary antibody, EnVision Flex HRP (Dako Omnis, Singapore) was dripped onto the samples and kept at room temperature for 20 min. After 20 min, 2 washes were performed with PBS. Immuno-localization EnVision Flex Substrate Working solution (50 μL DAB/Chromogen + 1 mL Substrate Buffer) was added to the samples and kept at room temperature for 5 min and washed with PBS. Then, samples were incubated with hematoxylin stain for 15 min and washed with deionized water and then PBS. Next, samples were incubated with eosin dye for 1.5 min and washed with PBS and the preparation was covered with glycerol. Eight random fields were photographed using 20× and 40× microscope (Olympus, Hamburg, Germany) magnifications. The stained and unstained cells were counted, proportioned, and statistically evaluated to obtain both quantitative and qualitative results.

### 2.5. Investigation of Morphology Characteristics of COC Cells by Hematoxylin and Eosin Staining

We prepared a smear of COC cells isolated from the FF of NOR and DOR cases’ MI and MII phases oocytes. The slides were fixed in 37% formaldehyde solution (Sigma Aldrich, Oakville, ON, Canada) for 15 min. After fixation, hematoxylin (Sigma Aldrich, Canada) dye was dripped onto the samples, which were incubated for 10 min and then washed with tap water. Afterwards, eosin stain (Sigma Aldrich, Canada) was dripped onto the slide, which was incubated for 1 min and washed with tap water. Finally, preparations were examined under a light microscope (Olympus, Germany) at 4×, 10× and 20× magnification and photographed.

### 2.6. Metadata Analysis: Female and Male Age, Oocyte Count, Oocyte Fertilization, and Embryo Development

The information of cases admitted to Istanbul Bahçeci Umut IVF Center between 22 April 2024 and 22 August 2025 was collected retrospectively. This retrospective information included female and male age, oocyte count, oocyte fertilization, and embryonic development.

### 2.7. Statistical Evaluations

All statistical evaluations were performed using the IBM SPSS Statistics 26.0 package program. Normal distribution of continuous variables was obtained using the Kolmogorov–Smirnov compatibility test. Comparison of quantitative non-normally advanced distribution, Bonferroni’s Correction test and independent samples of the Kruskal–Wallis test was applied. Comparisons between normally distributed variable groups were evaluated using Pearson’s correlation test. Comparisons between non-normally distributed variable groups were evaluated using the Kruskal–Wallis test. Spearman’s Rho correlation test was applied. Normal distribution analysis of retrospective data was evaluated using the Kolmogorov–Smirnov and Shapiro–Wilk tests. Multiple comparisons of female and male ages were evaluated using the Tukey HSD post hoc test. Non-parametric tests were applied for oocyte count and oocyte fertilization, the independent samples Kruskal–Wallis test was performed. Multiple comparisons of oocyte count and oocyte fertilization data were evaluated using the Benferroni test. Embryonic development assessment was evaluated using the Chi-square test. Correlations were determined using Spearman’s Rho test. The data obtained were written as mean ± standard deviation (SD) in the text. Significance levels between groups (*p* < 0.05; *p* < 0.01; and *p* < 0.001 or *p* > 0.05) were determined. Predictive modeling and diagnostic performance assessments were conducted for the logistic models, AUC estimation, and Youden-J-based metrics and we inserted the above findings into the Results Section (stratified analyses). A summary table of the threshold metrics and ROC figures for MI and MII were included in the Supplementary Materials (Supplementary Materials S2).

## 3. Results

The primary comparison exceeded the minimal detectable difference estimated a priori (see the Methods Section for details about the sample size and power).

### 3.1. Determination of CCNA2 and CCNB3 Gene Expression by Real-Time Polymerase Chain Reaction (qPCR-Based Assay)

**Figure 1:**
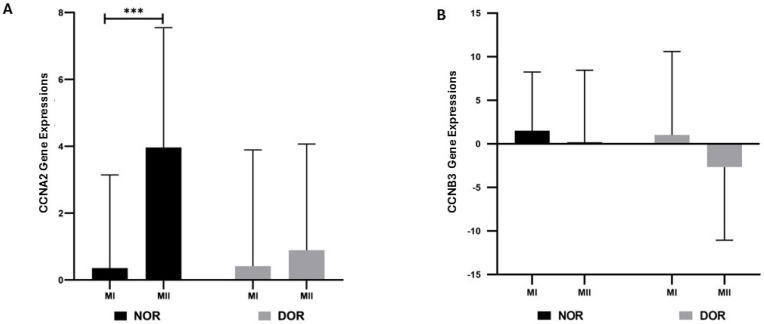
The gene expressions of CCNA2 and CCNB3 were determined based on normoresponder (NOR) and diminished ovarian reserve (DOR) cases’ MI and MII COC cells in oocyte phases [ns indicates a *p* > 0.05, which represents non-significance; and *** indicates *p* < 0.001; error bars represent the mean ± standard deviation (SD)]. (**A**) The CCNA2 gene expressions in the NOR and DOR cases’ MI and MII phases (*** *p* < 0.001; *p* > 0.05). (**B**) CCNB3 gene expressions in the NOR and DOR cases’ MI and MII phases (*p* > 0.05).

According to the data obtained, a statistically significant increase in CCNA2 gene expression was observed in NOR MII (3.962 ± 3.584) compared to the MI phase (0.354 ± 2.787) (*p* < 0.001; Figure 1). In the DOR MII phase (0.890 ± 3.175), no statistical increase was observed in CCNA2 gene expression compared to the MI phase (0.411 ± 3.479) (*p* > 0.05; Figure 1). The increase in CCNB3 gene expression in both the NOR MI phase (1.511 ± 6.727) compared to the MII phase (0.193 ± 8.260) and in the DOR MI phase (1.036 ± 9.560) compared to the MII phase (−2.656 ± 8.405) was not statistically significant (*p* > 0.05; Figure 1)

### 3.2. Determination of miRNA (miR-17, miR-106b, miR-190a, and miR-1275) Expressions by Real-Time Polymerase Chain Reaction (qPCR-Based Assay)

**Figure 2:**
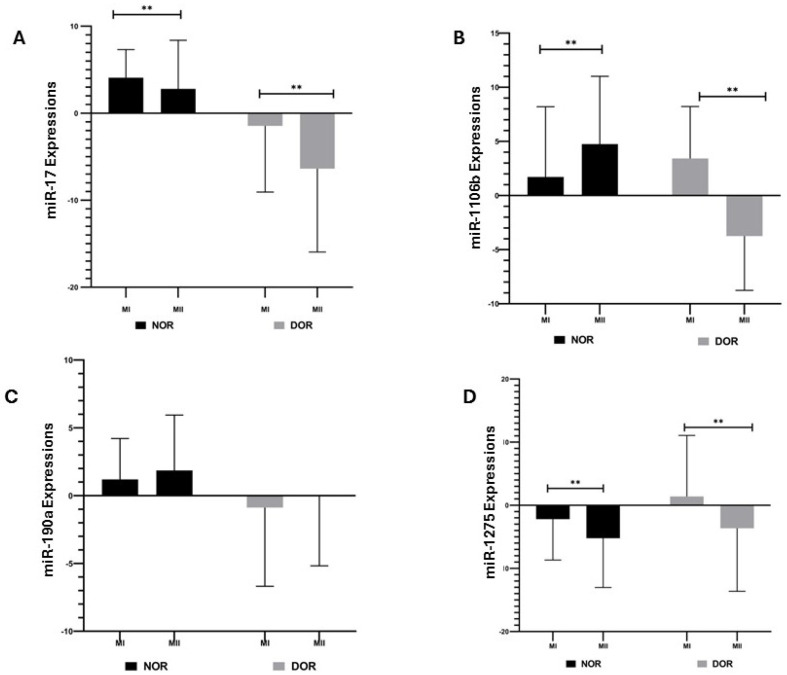
The expressions of miR-17, miR-106b, miR-190a, and miR-1275 were determined based on normoresponder (NOR) and diminished ovarian reserve (DOR) cases’ MI and MII phases in COC cells [ns indicates a *p* > 0.05, which represents non-significance and ** indicates a *p* < 0.05; error bars represent the mean ± standard deviation (SD)]. (**A**) miR-17 expressions in the NOR and DOR cases’ MI and MII phases (** *p* < 0.05); (**B**) miR-106b expressions in the NOR and DOR cases’ MI and MII phases (** *p* < 0.05); (**C**) miR-190a expressions in the NOR and DOR cases’ MI and MII phases (*p* > 0.05); (**D**) miR-1275 expressions in the NOR and DOR cases’ MI and MII phases (** *p* < 0.05).

miR-17 expression showed a statistically significant increase in the NOR MI phase (4.085 ± 3.226) compared to the MII phase (2.795 ± 5.588) (*p* < 0.05; Figure 2); similarly, the DOR group showed a statistically significant increase in the MI phase (−1.457 ± 7.606) compared to the MII phase (−6.368 ± 9.575) (*p* < 0.05; Figure 2). The miR-106b expression in the NOR group showed a statistically significant increase in the MII phase (4.754 ± 6.263) compared to the MI phase (1.719 ± 6.477) (*p* < 0.05; Figure 2); the DOR group showed a statistically significant increase in miR-106b expression in the MI phase (3.424 ± 4.799) compared to the MII phase (−3.749 ± 5.015) (*p* < 0.05; Figure 2). The increase in miR-190a expression in the NOR group MII phase (1.863 ± 4.077) compared to the MI phase (1.199 ± 3.017) was not statistically significant (*p* > 0.05; Figure 2); likewise, the increase in miR-190a expression in the DOR group MII phase (−0.036 ± 5.135) compared to the MI phase (−0.875 ± 5.808) was not statistically significant (*p* > 0.05; Figure 2). The increase in miR-1275 expression in the NOR group MI phase (−2.194 ± 6.482) compared to the MII phase (−5.221 ± 7.798) was statistically significant (*p* < 0.05; Figure 2); a statistically significant increase was also observed in the DOR group MI phase (1.394 ± 9.669) compared to the MII phase (−3.632 ± 9.988) (*p* < 0.05; Figure 2).

### 3.3. Investigation of CCNA2 and CCNB3 Protein Expression by Immunohistochemistry (IHC)

**Figure 3:**
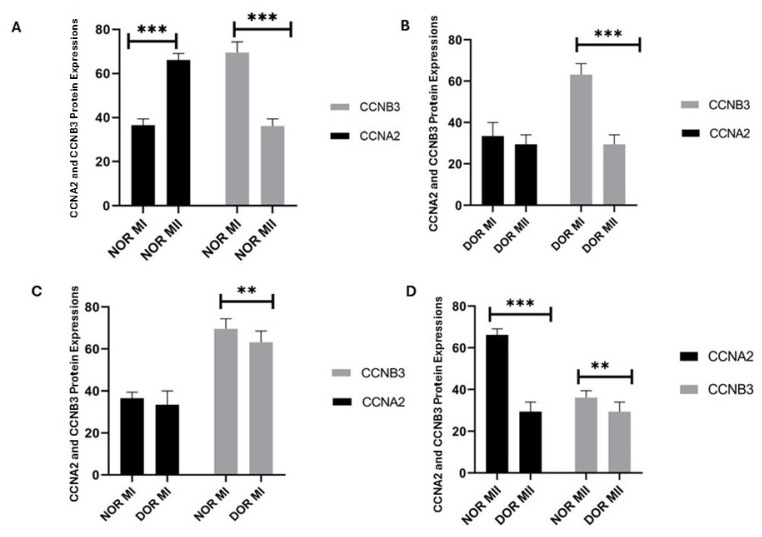
The determination of the CCNA2 and CCNB3 protein expression identified from normoresponder (NOR) and diminished ovarian reserve (DOR) cases’ MI and MII phases of oocytes’ COC cells by immunohistochemical analysis [ns indicates *p* > 0.05 as non-significance; ** indicates *p* < 0.05; and *** indicates *p* < 0.001; error bars represent mean ± standard deviation (SD)]. (**A**) The CCNA2 and CCNB3 protein expression in the NOR MI and MII phases (*** *p* < 0.001). (**B**) The CCNA2 and CCNB3 protein expression in the DOR MI and MII phases (*p* > 0.05; *** *p* < 0.001). (**C**) The CCNA2 and CCNB3 protein expression in the NOR MI and DOR MI phases (*p* > 0.05; ** *p* < 0.05) (**D**) The CCNA2 and CCNB3 protein expression in the NOR MII and DOR MII phases (*** *p* < 0.001; ** *p* < 0.05). [** *p* < 0.05 and *** *p* < 0.001; NOR: normoresponder (control) group, DOR: diminished ovarian reserve, MI: COC isolated from metaphase-I oocyte (immature oocyte); and MII: COC isolated from metaphase-II oocyte (mature oocyte)].

CCNA2 protein expression showed a statistically significant increase in the MII phase (66.142 ± 2.968) compared to the MI phase (36.571 ± 2.878) in the NOR group (*p* < 0.001; Figure 3). The CCNB3 protein expression in the NOR group showed a statistically significant increase in the MI phase (69.571 ± 4.825) compared to the MII phase (36.142 ± 3.287) (*p* < 0.001; Figure 3). The CCNA2 protein expression was not statistically significant in the MII phase (29.428 ± 4.613) of the DOR group compared to the MI phase (33.428 ± 6.553) (*p* > 0.05; Figure 3), whereas CCNB3 protein expression showed a statistically highly significant increase in the MI phase (63.142 ± 5.336) of the DOR group compared to the MII phase (29.428 ± 4.613) (*p* < 0.001; Figure 3). The CCNA2 protein expression was not statistically significant in the NOR group MI phase (36.571 ± 2.878) compared to the DOR group MI phase (33.428 ± 6.553) (*p* > 0.05; Figure 3). The CCNB3 protein expression in the NOR group MI phase (69.571 ± 4.825) showed statistically lower significance compared to the DOR group MI phase (63.142 ± 5.336) (*p* < 0.05; Figure 3). The CCNA2 protein expression showed a statistically highly significant increase in NOR group MII phase (66.142 ± 2.968) compared to the DOR MII phase (29.428 ± 4.613) (*p* < 0.001; Figure 3). The CCNB3 protein expression showed statistically lower significance in the NOR group MII phase (36.142 ± 3.287) compared to the DOR MII phase (29.428 ± 4.613) (*p* < 0.05; Figure 3). The values of protein expression are indicated as the percentage of positive cells.

### 3.4. Determination of Morphology Characteristics of COC Cells by Hematoxylin and Eosin Staining

**Figure 4:**
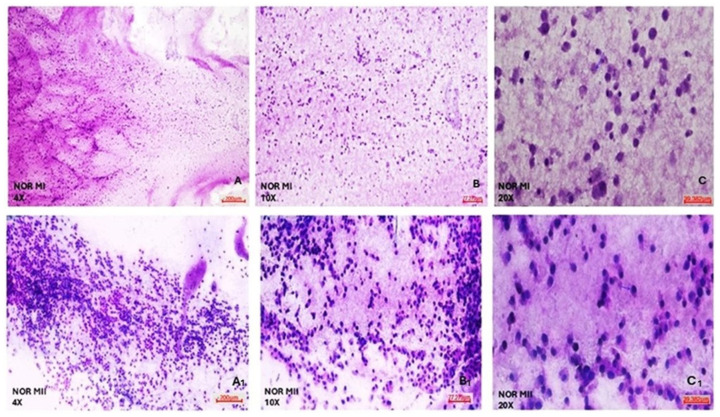
Morphological evaluation of NOR MI and MII phases of oocytes’ COC cells. (**A**) NOR MI 4×; (**B**) NOR MI 10×; and (**C**) NOR MI 20× magnification. (**A_1_**) NOR MII 4×; (**B_1_**) NOR MII 10×; and (**C_1_**) NOR MII 20× magnification of the light microscope.

According to the morphological analyses of COC cell populations of MI and MII phase oocytes of NOR and DOR patient groups conducted via hematoxylin and eosin staining, NOR group MI phase COC cells generally exhibited a round shape. Most of the nuclei were oval, but some nuclei had a polygonal appearance. The cell cytoplasm was of a normal size, but the cell density decreased. Furthermore, both the nuclei and cytoplasm of COC cells were sparsely stained (Figure 4). According to the MII phase COC cells of the NOR group, almost all the cells exhibited a round shape, and most of the nuclei had an enlarged polygonal appearance and showed an increase in the density of the cells, with a decreased cytoplasm size observed. Both the nuclei and cytoplasm of COC cells were intensively stained with hematoxylin and eosin stain (Figure 4).

**Figure 5:**
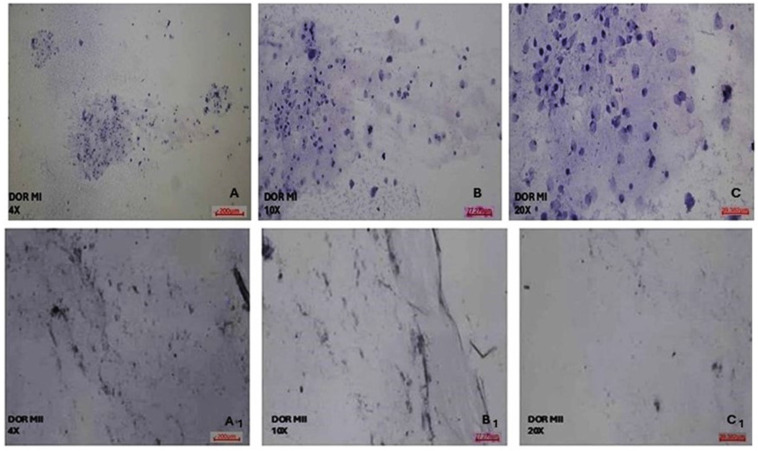
Morphological evaluation of DOR MI and MII phases of oocytes’ COC cells. (**A**) DOR MI 4×; (**B**) DOR MI 10×; and (**C**) DOR MI 20× magnification. (**A_1_**) DOR MII 4×; (**B_1_**) DOR MII 10×; (**C_1_**) DOR MII 20× magnification of light microscope.

It was observed that DOR group MI phase COC cells generally exhibited a round shape, but the cell size was reduced. Most of the nuclei had an oval appearance, and there was a great reduction in cell cytoplasm and density. In addition, both the nucleus and cytoplasm of COC cells were barely stained with hematoxylin and eosin stain (Figure 5). In the DOR group MII phase COC cells, almost all the cells were round, most of the nuclei were small and located close to the periphery, no change was observed in the size of the cytoplasm, and the density of the cells was greatly reduced. Both nucleus and cytoplasm of COC cells were almost completely unstained after treatment with hematoxylin and eosin stain (Figure 5).

### 3.5. Metadata Analysis: Female and Male Age, Oocyte Level, Oocyte Fertilization, and Embryo Development

The metadata (female and male ages, collected number of oocytes, oocyte fertilization rate, and embryo development) of the cases referred to Istanbul Bahçeci Umut IVF Centre between 22 April 2024 and 1 August 2025 were collected retrospectively.

**Figure 6:**
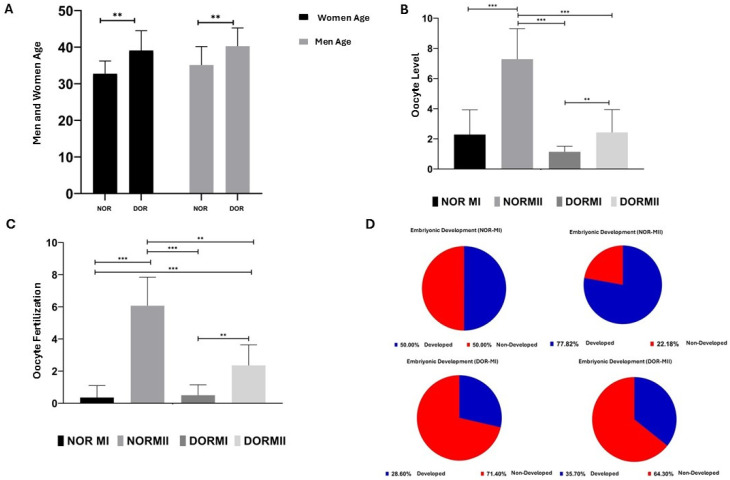
Metadata analysis of the NOR and DOR cases, performed retrospectively [ns = *p* > 0.05, which indicates non-significance; ** represents a *p* < 0.05; and *** represents a *p* < 0.001; error bars represent the mean ± standard deviation (SD)]. (**A**) The female and male NOR and DOR case groups (** *p* < 0.05); (**B**) oocyte-level analysis in the NOR MI, MII and DOR MI, and MII cases (*** *p* < 0.001; *p* > 0.05); (**C**) the oocyte fertilization in the NOR MI, MII and DOR MI, and MII cases. (** *p* < 0.05; *** *p* < 0.001); (**D**) embryo development potential in the NOR MI, MII and DOR MI, and MII cases (50% in NOR MI, 77.8% in NOR MII, 28.6% in DOR MI, and 35.7% in DOR MII).

Of the NOR and DOR cases analyzed within the female age group, the increase in the DOR group (39.07 ± 5.441) compared to the NOR group (32.71 ± 3.517) was statistically significant (*p* < 0.05, Figure 6). In the NOR and DOR cases from the male age group, a statistically significant increase in the DOR (40.28 ± 4.983) group compared to the NOR (35.14 ± 5.036) group was observed (*p* < 0.05, Figure 6). A statistically significant increase in oocyte-level analysis was observed in the NOR MII group (7.28± 2.016) compared to both the NOR MI (2.28± 1.637) and DOR MII (2.42± 1.504) groups (*p* < 0.001, Figure 6). No statistically significant increase was observed in the DOR MII (2.42 ± 1.504) group compared to the NOR MI (2.28 ± 1.637) group (*p* > 0.05, Figure 6). The increase in the NOR MI (2.28 ± 1.637) group compared to the DOR MI (1.14 ± 0.363) group was not statistically significant (*p* > 0.05, Figure 6). The increase in DOR MII (2.42 ± 1.504) (*p* < 0.05, Figure 6) and NOR MII (7.28 ± 2.016) groups compared to DOR MI (1.14 ± 0.363) group was statistically significant (*p* < 0.001, Figure 6). A comparison of oocyte fertilization rates among the NOR MI, NOR MII, DOR MI, and DOR MII groups revealed that the increase observed in the DOR MI group (0.50 ± 0.650) relative to the NOR MI group (0.35 ± 0.744) was not statistically significant (*p* < 0.05, Figure 6). A statistically significant increase was observed in DOR MII (2.35 ± 1.277) and NOR MII (6.07 ± 1.774) groups compared to the NOR MI (0.35 ± 0.744) group (*p* < 0.001, Figure 6). Compared to the DOR MI (0.50 ± 0.650) group, the increases in the NOR MII (6.07± 1.774) (*p* < 0.001, Figure 6) and DOR MII (2.35 ± 1.277) (*p* < 0.05, Figure 6) groups were statistically significant. The increase in the NOR MII (7.28 ± 2.016) group compared to the DOR MII (1.14 ± 0.363) group was not statistically significant (*p* < 0.05, Figure 6). We also observed that the post-fertilization embryonic development rate of the oocytes of the collected COC samples was 50% for NOR MI, 77.8% for NOR MII, 28.6% for DOR MI, and 35.7% for the DOR MII cases (Figure 6).

## 4. Discussion

The maturation of oocytes involves intricate and extended processes such as folliculogenesis and oogenesis, which together establish the foundation necessary for early embryonic development. These multistep developmental mechanisms are characterized by specific transcriptional mechanisms and associated with important transcriptomic transitions [37]. The oocyte transcriptome is a dynamic, tightly controlled process leading to oocyte growth and maturation [38]. The primordial stage reflects strong communication between the oocyte and surrounding cells, with increased activation of genes involved in cell adhesion, epithelial cell proliferation, and collagen fibril organization pathways, but this activation tends to decrease as oocyte development continues [39]. The downregulation of cytoplasmic translation in antral follicles and the fact that the highest activity level is observed in primordial follicles supports the motion that maternal mRNAs are not progressively translated but are stored until they are translated as meiosis resumes following the germinal vesicle breakdown (GVBD) stage [40]. Accordingly, the factors related to the paracrine communication between the oocyte and the granulosa cells play important roles in oocyte development and maturation. Recent molecular research on germ cells and reproductive mechanisms has demonstrated that miRNAs play essential roles as master regulators in the post-transcriptional and translational repression of target gene mRNAs, thereby influencing key processes such as follicle development and oocyte maturation. [41,42]. Unlike in somatic cells, the completion of meiosis I and meiosis II that allows the oocyte to become a haploid gamete is the crucial and final step in the maturation process in oogenesis [26]. The progression of meiosis involves two unique meiotic intervals and resumption of the cell cycle from the interrupted phase is regulated specifically to the MI and MII phases depending on the dynamic relationship between the cyclins and their catalytic subunits cdks [11,43]. The quality of the surrounding follicle microenvironment and the continuity of mutual cell communication are essential for the proper and healthy completion of oocyte development [8,44].

Within molecular parameters for oocyte maturity assessment, cumulus oophorous complex (COC) cells provide a valuable opportunity for data collection. According to the literature, many studies have evaluated the expression levels of selected oocyte development-related genes in COC cells in relation to oocyte maturation [12], embryo development capacity followed by sac formation [13], pregnancy follow-up [45], and live birth rates [14].

Diminished ovarian reserve (DOR) refers not only to a reduced number of follicles in the ovary, but also to the quality of oocytes, and it negatively affects fertility in women [46]. Decreased oocyte quality leads to an increased incidence of abnormal meiotic aneuploidy during oocyte development [47], mitochondrial dysfunction [48], DNA damage [49], and failed oocyte maturation due to asynchronous development of the cytoplasm and nucleus [50]. Dysfunctions in the cumulus oophorous complex (COC) cells and granulosa cells of DOR patients’ oocytes may be at the transcriptional level, because there are significant differences between the mRNA expression profiles according to the normoresponder (NOR) oocytes [51].

Cyclins play different roles in the meiosis-specific regulation of germ cells. A study in male and female mice with the CCNB3 mutation suggested that the CCNB3 gene functions only in female meiosis, which may be related to the prolonged interruption of meiosis I, reflecting different mechanisms of meiotic regulation between the two sexes [52]. While previous studies indicated that the CCNB3 gene regulates the meiotic cell cycle in Drosophila [11,53], recent studies in mice reported that CCNB3 also plays an important role during oocyte maturation and inhibits the CCNB3 gene in oocytes, leading to MI arrest [54,55]. Oocytes with an inhibited CCNB3 gene are characterized by insufficient anaphase promoting complex/cyclosome (APC/C) activation and reduced cyclin B1-cdk1 complex activity [56]. The increase in CCNB3 gene expression level in the NOR MI phase and DOR MI phase compared to MII was a partially expected result. However, the standard deviation of the CCNB3 gene was very high, suggesting that increasing the number of samples may reduce the standard deviation and better reflect the expected results. While CCNB3 gene expression initiates anaphase during meiosis I and promotes the degradation of APC/C substrates, these findings suggest that CCNB3 is not suitable as evidence for MI phase identification in NOR and DOR cases.

A study investigating CCNB1 and CCNA2 gene inhibition in mice found that both CCNB1-null and CCNA2-null mice exhibited infertility. Notably, CCNB1-null oocytes were able to complete the metaphase I (MI) stage but subsequently arrested during the interphase. On the other hand, CCNA2-null oocytes showed no significant delay in meiosis I progression, but exhibit impaired kinetochore–microtubule attachment due to delays in MII spindle formation [57]. Zang et al. showed that CCNA2-null oocytes failed to complete the MII phase and PB formation reduced, but even 31.7% of the oocytes with discarded PB had defects in the anaphase and telophase cell division, despite undergoing sister chromatin segregation. However, most CCNA2-null oocytes (75.4%) showed a delayed chromosome structure in anaphase compared to the control (16%). These results confirm that CCNA2 plays an important role in spindle and chromosome organization, especially during the MII phase completion of meiosis [30]. The high expression of the CCNA2 gene in the MII compared to the MI in NOR group, the selection of MII oocytes based on their morphology indicating their maturation, and the high fertilization capacity suggest that the CCNA2 gene has the potential to be evidence for the NOR group MII. The high CCNA2 protein expression in the MII phase in NOR group, which tends to complete the oocyte maturation process without the influence of genetic or environmental factors such as DOR, indicates that the CCNA2 gene promotes the transition to the MII phase and supports the completion of the MII phase. Therefore, CCNA2 shows potential as evidence for protein expression in the MII phase of the NOR group. Although CCNA2 gene expression in the DOR MII group increased according to the MI, the increase was not statistically significant. As a result, DOR is characterized by a decrease in oocyte volume and quality; the number of oocytes is limited to between two and six, although the collected MII oocytes that have mature morphology do not show genetically sufficient oocyte development potential. Moreover, poor oocyte quality is generally related to low oocyte numbers, chromatin disintegration, chromosome decondensation, and nuclear abnormalities and these failures lead to abnormal gametogenesis, fertilization, implantation, and early development of the embryo. Therefore, molecular changes in COC cells surrounding oocytes in cases with DOR indicate that the CCNA2 gene cannot be used as evidence for DOR MII phase prediction.

According to the analysis, CCNB3 protein expression showed a high increase in NOR MI compared to MII, since the CCNB3 gene promotes the completion of MI phase by triggering the onset of anaphase in the first meiosis by stimulating degradation of APC/C substrates. Therefore, the results indicate the potential of CCNB3 as evidence in terms of protein expression for MI phase prediction in the NOR group. CCNB3 protein expression showed a statistically significant increase in DOR MI compared with MII phase. The high expression profile of CCNB3 protein in MI phase is an expected result, because the CCNB3 gene triggers the onset of anaphase in the first meiosis and promotes the completion of the MI phase; moreover, the oocytes of the DOR group generally arrested in the MI phase. As a result, the high CCNB3 protein expression profile in the MI phase of both NOR and DOR groups indicated that CCNB3 has the potential to provide evidence of protein expression in both the NOR and DOR MI phase.

MicroRNAs play a crucial role in post-transcriptional regulation and influence numerous biological processes such as oocyte development, maturation, fertilization, implantation, and early embryonic development. Recent research indicates that miRNA expression can be modulated by both overexpression and inhibition mechanisms [58,59,60]. The regulation of gene expression is crucial for oocyte development, as changes in expression can result in suboptimal oocyte quality [61]. Gene expressions vary depending on the dynamic expression levels of the miRNA-17-92 cluster, which is among the short non-coding RNA molecules that regulate granulosa cells’ functions. The high expression in the miR-17-92 cluster promotes granulosa cell proliferation and reduces the number of differentiated cells, while its inhibition decreases granulosa cell proliferation and increases differentiation [62]. In another study, the high expression profile of miR-17-5p in porcine granulosa cells (pGCs) led to a decrease in the luciferase activity of E2F transcription factor 1 (E2F1) and increased cell growth, as well as the expression of genes involved in follicular development such as luteinizing hormone receptor (LHR), the cytochrome P450 19A1 (CYP19A1) gene, amphiregulin (AREG), and estradiol synthesis. On the other hand, inhibition of miR-17-5p expression suppressed cell growth, follicular development of marker genes’ expression, and estradiol synthesis [63]. The transcription factor E2F1 is an important cell cycle regulator and targets genes that encode proteins for the regulation of cell cycle progression through the G1/S transition and DNA repair mechanisms and apoptosis [64]. The high expression of miR-17 in NOR MI compared to MII clearly indicates that NOR MI phase granulosa cells increase their potential to transition MII by promoting proliferation and moreover, miR-17 regulates cell cycle progression through G1/S transition. Similarly, miR-17 expression in the MI phase is higher than in the MII phase of DOR; it diminishes the number of follicles in the ovary and reduces the quality of oocytes, and it adversely affects fertility. The developmental potential of oocytes in DOR cases is significantly lower according to that noted in NOR cases, which indicates that miR-17 is involved in the completion of the DOR MI phase, but despite this, there are problems in the completion of maturation even if the oocyte passes to the MII phase due to the fact that it leads to unsuccessful oocyte maturation in many respects. This may take the form of low oocyte quality; increased incidence of abnormal meiotic aneuploidy in developmental stages; DNA damage; and asynchronous development of the cytoplasm and nucleus. Therefore, the high level of miR-17 expression in the NOR and DOR group MI compared to MII oocytes suggests that miR-17 can be used as evidence in the transition from the MI to MII phase.

Oocytes contain a variety of somatic cell types in their surroundings, such as granulosa, theca, and the COC cells [65], and several miRNAs have been identified as regulators of intra-ovarian gene expression for the healthy progression of complex physiological processes such as folliculogenesis and steroidogenesis [66]. Studies have reported that differential expression of miRNAs is associated with ovarian diseases such as ovarian cancer, polycystic ovary syndrome (PCOS), and premature ovarian failure (POF) [67,68]. Shapira et al. evaluated the miRNA profiles in plasma samples collected preoperatively from cases with serous epithelial ovarian cancer and benign neoplasms. As a result, 22 miRNAs were shown to be differentially expressed between the controls and ovarian cancer. Six of these miRNA profiles (miR-106b, miR-126, miR-150, miR-17, miR-20a and miR-92a) could be distinguished in benign and malignant ovarian cancer cases [69]. While significantly different expression levels of miR-106b, miR-126, miR-150, miR-17, miR-20a, and miR-92a were observed in benign neoplasm groups, it has been previously reported that, in particular, miR-106b, miR-150, and miR-126 have decreased expression in ovarian cancer tissue compared to non-cancerous ovarian tissue [70,71]. miR-106b cluster (-106a/b, -17-5p, -20a/b, and -93) show high expression levels in many cancer tissues and they promote cell cycle progression, while loss of function reverses this phenotype [72]. Furthermore, another study showed that miR-106b cluster members play a functional role in the cell cycle and target the cdk inhibitor p21 by promoting the transition from G1 to S phase [73]. The high expression of miR-106b in the NOR MII compared to the MI phase indicated that miR-106b promoted the transition to the MII phase by regulating the completion of the MI phase in the NOR group. On the other hand, the high expression profile of miR-106b in DOR MI compared to MII suggests that a defect in the targeted gene by miR-106b may cause the cells to remain in the MI phase and cause disruptions in the G1/S transition. miR-106b upregulation may have the potential to be used in the determination of oocyte developmental competence for the MII phase by supporting the transition to the MII phase in NOR group cells. High expression of miR-106b was observed in the DOR patient group MI phase cells compared to the MII phase cells. miR-106b prevents the transition to the MII phase and causes them to remain in MI phase, indicating that miR-106b can be used for the determination of the MI phase in the DOR patient group. Thus, miR-106b upregulation in NOR MII has the potential to be used in the prediction of the oocyte developmental stage for the MII phase in the NOR group. We observed high expression of miR-106b in DOR patient group MI phase cells compared to MII phase cells. miR-106b prevents the transition to the MII phase and causes them to remain in the MI phase, indicating that miR-106b can be used for the determination of the MI phase in the DOR patient group. In the DOR cases, the high expression of miR-106b in the MI compared to the MII phase suggests that miR-106b may be useful for the determination of the MI phase in DOR cases by causing arrests in the MI phase.

Most follicles at different stages of follicular development undergo a degenerative process known as atresia, such that approximately <1% of follicles may complete ovulation [74,75]. Granulosa cells are the main target cells in studies that aim to understand the role of epigenetic regulatory factors, including miRNAs, in order to identify the mechanism of follicular atresia [76,77]. While the estrogen (E2) hormone protects granulosa cells from apoptosis [78] and promotes their proliferation [79], high doses of E2 suppress their proliferation [80]. Studies have shown that follicle-stimulating hormone (FSH) is required for E2 hormone production [81] and induces the expression of CYP19A1 [82,83]. In addition, various epigenetic mechanisms, such as those mediated by non-coding RNAs, play a vital role in E2 hormone synthesis [84,85]. Liu et al. showed that miR-1275 expression was upregulated during follicular atresia in pig ovaries [86], and miR-1275 induced early apoptosis in pGCs by targeting the CYP19A1 gene and decreased E2 hormone synthesis by initiating follicular atresia [23]. According to the data, the miR-1275 expression was higher in the NOR MI compared to the MII phase. This is supported by a study showing that miR-1275 prevents the transition to the MII phase by regulating early apoptosis in the NOR MI phase and causes arrest in the MI phase. Likewise, miR-1275 showed a higher expression profile in the DOR MI compared to the MII phase with DOR cases, indicating that apoptotic processes occur at different stages of oocyte development, starting at the follicular stage and affecting both oocyte reserve and development. Therefore, the high expression profile of miR-1275 in both the NOR and DOR MI phase suggests that miR-1275 can be used for MI phase prediction in the NOR and DOR cases group in terms of evaluating oocyte maturation.

Although there is great variation in the oocytes reserve in each woman, our knowledge about the factors controlling the oocyte reserve is limited. Even though it is a normal physiological condition that oocyte quantity and quality decrease with increasing female age, some women face DOR cases much earlier than expected and become infertile [87]. The main clinical features of DOR cases are characterized by the presence of regular menstrual periods and abnormal ovarian reserve in the pre-menopausal period [88]. On the other hand, premature ovarian failure (POF) is a gynecological disorder of uncertain etiology which causes infertility in women of reproductive age. As another important pathogenic factor, it is diagnosed by postmenopausal FSH levels and four or more months of secondary amenorrhea in women under the age of 40 [89]. With the deepening of molecular research, it has been confirmed that overactivation of primordial follicles is an important pathological mechanism for POF and is also caused by the early depletion of ovarian reserves [90,91,92]. To further investigate the role of miRNAs in oocyte development and maturation, Halvei et al. used miRNA sequencing to characterize miRNA populations in possible zygote pools in bovine germinal vesicle (GV) oocytes and MII oocytes and found a defined miRNA population at each stage of maturation [21]. In another study conducted by Yao et al., significant differential changes occurred in the miRNA-190a-5p expression profile after 4-vinylcyclohexene diepoxide (VCD) was administered to the ovaries of rats with premature ovarian failure (POF), and the miRNA-190a-5p expression profile showed an upregulated propensity from the sixth day of VCD treatment. As a result, miRNA-190a-5p can offer potential evidence for early screening of POF and can continuously activate primordial follicles in rats by targeting the expression of PHLPP1 gene and key proteins in AKT-FOXO3a and AKT-LH/LHR pathways [18]. According to this study, the miR-190a expressions showed no statistical difference between MI and MII phases in both the NOR and DOR cases (*p* > 0.05). Considering the data obtained from previous studies, it has been shown that miRNA-190a-5 causes hyperactivation of primordial follicles in rats and promotes POF, directly or indirectly causing abnormal levels of LH hormone and decreased LHR expression. Although miRNA-190a-5p has been shown to be potential evidence for early screening of POF, it is expected that miRNA-190a shows a different expression profile in the DOR cases, which may be due to the dynamic changes in the expression profiles of miRNAs. On the other hand, follicular cell proliferation, which proceeds synchronously with oocyte development, results in differentiation of primordial follicle cells into granulosa and cumulus cells. Therefore, miR-190a induced hyperactivation in primordial follicles, whereas miR-190a expression was not observed in COC cells, in contrast to primordial follicles in granulosa and cumulus cells in the MI and MII phases of both NOR and DOR cases. Therefore, although miR-190a showed a higher expression profile in the MII phase in the NOR and DOR groups compared to the MI phase, it was not statistically significant, which indicates that it cannot be used for the prediction of the MII phase in NOR or DOR cases.

According to the correlation analysis, the CCNA2 gene in NOR MI phase was positively correlated with miR-17 (r < 0.01), miR-1275 (r < 0.05), miR-106b (r < 0.05) and miR-190a (r < 0.01) in the NOR MII and DOR MII phases. In the DOR MI phase, CCNA2 showed a positive correlation with CCNB3 (r < 0.05). CCNB3 in NOR MI, NOR MII, and DOR MII revealed no correlation with any miRNA (r > 0.05) or CCNA2 (r > 0.05). On the other hand, CCNB3 showed negative correlation with CCNA2 in DOR MI (r < 0.05). miR-190a showed no correlation with any miRNAs (r > 0.05) or CCNB3 and CCNA2 genes (r > 0.05) in NOR MI and miR-17 positively correlated with miR-190a (r < 0.05) in the DOR MI group.

To better characterize the functional role of COC cell populations in the MI and MII phases of the NOR and DOR cases, morphological analysis of cell cytoplasm, nuclear structure, and cell density was investigated via hematoxylin and eosin staining. The morphological characteristics of the MII phase COC cells in the NOR group were better than in the MI; this was the expected result for the MII phase in the NOR group. The mature morphological features of the oocytes selected showed a clear cytoplasm, a single PB, an appropriate ZP thickness and PV space, and an increase in the number of COC cells, which indicated the oocyte quality. On the other hand, the morphological characteristics of the DOR MI group are much better than those of MII, because the COC morphology is related to the degree of atresia of the follicle that forms it. This insufficiency in their capacity to transition from the MI to the MII phase negatively affects both the proliferation and differentiation of the follicles. Moreover, it is known that failures occur in implantation, embryonic development, and pregnancy rates even if the fertilization of DOR MI or MII oocytes is achieved.

NOR and DOR groups who applied to Istanbul Bahçeci Umut IVF Center between 22 April 2024 and 22 August 2025 had their metadata collected. This included female and male age, oocyte count, oocyte fertilization rate, and embryo development potential; these factors were evaluated retrospectively. Regarding the mean of the male and female ages of the patient groups participating in the study, no statistically significant difference was found in either the NOR or DOR cases (*p* > 0.05). This result demonstrates that the age factor had no impact on the results obtained when evaluating the other study parameters and the collected samples were appropriate in terms of ages. The increase in the number of oocytes collected for NOR MII compared to the NOR MI and DOR MII groups was highly significant (*p* < 0.001). The main reason why the NOR MII group has the highest number of oocytes compared to others is that this group generally reveals normal developmental potential of meiosis division in maturation and usually has normal reproductive physiology compared to DOR cases. On the other hand, the higher oocyte counts in the DOR MII (*p* < 0.05) and NOR MII (*p* < 0.001) phases compared to DOR MI were expected. This is due to the lower number of oocytes produced in the ovaries of the DOR patient group and the lower oocyte development and maturation capacity, which support the idea that fewer oocytes are produced compared to in the NOR MII phase. In addition, the higher number of oocytes collected in the DOR MII (*p* < 0.05) compared to the DOR MI phase suggests that the failed transition to MII was due to the inadequate developmental capacity of the DOR MI oocytes. When comparing the fertilization rates of NOR and DOR oocytes, it is expected that the fertilization rate will be significantly higher in the NOR MII (*p* < 0.001) and DOR MII (*p* < 0.001) groups compared to the NOR MI phase. Similarly, there was a significant increase in fertilization rates observed in NOR MII (*p* < 0.001) and DOR MII (*p* < 0.05) compared to DOR MI. This is because both NOR MI and DOR MI phase oocytes failed to reach their developmental potential and thus failed to mature, resulting in lower fertilization rates compared to mature oocytes in the DOR MII and NOR MII groups. The fertilization rates of NOR and DOR oocytes were significantly higher in the NOR MII (*p* < 0.001) and DOR MII (*p* < 0.001) groups compared to the NOR MI group. Similarly, a significant increase in fertilization rates was observed in the NOR MII (*p* < 0.001) and DOR MII (*p* < 0.05) groups compared to the DOR MI group.

NOR MI and DOR MI phase oocytes had lower fertilization and embryonic development rates than mature NOR MII and DOR MII oocytes. Embryonic development was completed in 50% of NOR MI, 78.6% of NOR MII, 28.6% of DOR MI, and 35.7% of DOR MII cases. It is expected that the embryonic development capacity is at its highest level in NOR MII and then DOR MII oocytes. Among the most fundamental reasons for this is that mature MII oocytes, with full nuclear maturation, demonstrate higher developmental potential compared to MI oocytes. On the other hand, the high expression of the CCNA2 gene in the NOR MII group correlates with high fertilization capacity and embryonic development capacity, indicating that it has the potential to provide evidence for the NOR MII group. Moreover, the NOR MI oocytes have strong potential to transition to the MII phase and their capacity to complete the maturation process is higher than that of DOR MI oocytes. CCNB3 protein exhibits a high expression profile in NOR MI phase compared to DOR MI, which supports the idea that NOR MII oocytes have a greater embryonic developmental capacity according to the DOR MII oocytes. CCNB3 is also known to play an important role during oocyte maturation and inhibition of the CCNB3 gene is characterized by inadequate anaphase promoting complex/cyclosome (APC/C) activation and leading to MI phase arrest. Based on the data, CCNB3 gene expression was not suitable for MI phase prediction in the NOR and DOR groups, despite its role in initiating anaphase during the first meiotic division of oocytes. The significance of the expected result in CCNB3 protein expression lies in the fact that proteins are the cell’s fundamental structural units and final gene products; thus, translation provides greater insight than transcription, especially given epigenetic influences like DNA methylation and histone modifications. The high CCNA2 gene expression in NOR MII demonstrates the potential usage of the CCNA2 gene as evidence for determining the MII in NOR cases. Especially, the significant expression of CCNA2 protein in NOR MII contributes to the accuracy of using CCNA2 as evidence in predicting the transition to the MII phase. The higher miR-17 expression in the NOR MI and DOR MI phases compared to the MII phase supports the results obtained from the literature, showing that the high miR-17 expression in the COC cells of MI phase oocytes in both NOR and DOR patients compared to MII phases indicates that miR-17 epigenetically regulates the completion of the MI phase and the transition to the MII phase. On the other hand, the high miR-106b expression in the NOR MII phase suggests that miR-106b may be an epigenetic regulator of oocyte developmental competence in the NOR MII phase by promoting the development of MII phase while it inhibits the transition to the MII phase in the DOR case and causes cells to remain in the MI phase. As a result, miR-106b may be an epigenetic regulator of the MI phase in the DOR cases compared to the NOR cases. The high miR-1275 expression in the MI phase of the NOR and DOR cases compared to the MII phase shows that apoptotic processes that occur at different stages of oocyte development begin at the follicular stage and affect both the size of the reserve and oocyte development. The results indicate that miR-1275 has the potential to be an epigenetic regulator of the MI phase in both NOR and DOR cases. Although miR-190a is considered potential evidence for a POF diagnosis, it is not suitable for determining MII phase maturation in NOR and DOR cases because the expression levels of miRNAs vary with different syndromes or cases.

## 5. Conclusions

Comparing the expression levels of CCNB3 gene for the prediction of the MI phase and CCNA2 gene for the determination of the MII phase, and also the profiling miR-17, miR-106b, miR-190a, and miR-1275 expression levels which target these genes, increases the importance of this study in terms of investigating the meiotic process in germ cells and their roles in fertilization capacity, since there are no genetic or epigenetic markers that clearly determine the transition from MI to MII among the existing studies in the literature.

Gaining insight into the molecular processes behind oocyte maturation in IVF clinics is essential for accurate diagnosis and effective treatment. According to the data obtained in this study, COC cells isolated from follicular fluid demonstrate significant potential and require prospective validation through clinical performance evaluation to assess their utility in early genetic diagnosis of oocytes in the MI or MII phases. While data on the follicles of individual patients from IVF centers was not available, researchers can differentiate between NOR and DOR groups by examining how CCNA2 and CCNB3 gene expression relate to their corresponding miRNAs. The determination of oocyte maturation in both NOR and DOR cases can be assessed by evaluating whether oocytes remain in the MI phase or progress to the MII phase, taking into account the correlations between CCNA2 and CCNB3 genes and relevant miRNAs, as well as by identifying the expression profiles of miRNAs that target the CCNB3 and CCNA2 genes, as well as those associated with these genes during the MI and MII phases.

The ability to examine the gene and protein expression levels used in various molecular pathways and their associated regulators, miRNAs, through genetic analysis provides comprehensive preliminary data to help us learn about the MI and MII stages of oocyte development and their fertilization capacity and embryonic development after completing maturation.

## Figures and Tables

**Table 1:** Forward and reverse/universal primer sequences used in (qPCR-based assay).

Accession Number	Genes/miRNAs	Forward Primer	Reverse Primer/Universal	Tm (Basic)
NG-007073.2	GAPDH	5′-CGAGGGGGGAGCCAAAAGGG-′3	3′-GAAACTGCGACCCCGACCGT-′5	55 °C
NM-001237	CCNA2	5′-CACTCTACACAGTCACGGGA-′3	5′-AGTGTCTCTGGTGGGTTGAG-′3	59 °C
NM-033670	CCNB3	5′-GCGAATACATCCCTGCCTTG-′3	5′-TTCGTTTGGCAATTCTCCCC-′3	59 °C
M10000071	miR-17	5′-ACTACCTGCACTGTAAGCACTTTG-3′	5′-CTTCTTATGGAGCCTGGGACTCTGACC-′3	61 °C
M10000734	miR-106b	5′-ACTGCTAAAGTGCTGACAGTGCA-′3	5′-CTTCTTATGGAGCCTGGGACTCTGACC-′3	61 °C
M10000486	miR-190a	5′-CGTGATATGTTTGATATATTAGGT-′3	5′-CTTCTTATGGAGCCTGGGACTCTGACC-′3	54 °C
M10006415	miR-1275	5’-ACACTCCAGCTCAGGTGGGGGAGAGGCTGTC-′3	5′-CTTCTTATGGAGCCTGGGACTCTGACC-′3	75 °C

## Data Availability

The original contributions presented in this study are included in the article/Supplementary Material. Further inquiries can be directed to the corresponding author.

## Supplementary Materials

The following supporting information can be downloaded at https://www.mdpi.com/article/10.3390/diagnostics15202658/s1, S1: Power and Sample Size Justification; S2: The Features of the Samples; S3: Stratified Diagnostic-Performance Analysis.

## Abbreviations

The following abbreviations are used in this manuscript:

FF	Follicular Fluid
COC	Cumulus Oophorous Complex
MPF	Maturation-Promoting Factor
OPU	Oocyte Pick Up
