# Supplementary material for: CCNA2 and CCNB3 as Early Potential Molecular Candidates of Oocyte Maturation in Cumulus-Oophorous Complex Cells from Follicular Fluid
Source: Diagnostics (Basel). 2025 Oct 21;15(20):2658. doi: 10.3390/diagnostics15202658 (PMC12563169; doi:10.3390/diagnostics15202658)
Supplement: Supplementary file 1 [file diagnostics-15-02658-s001.zip › diagnostics-3905723-supplementary.pdf]

Supplement Materials 1 (S1): Power and Sample Size Justification)

For sample-size justification, we focused on the clinically most relevant contrast in our design: the MI vs MII difference in CCNA2 ΔCt within the NOR stratum. In the results, the NOR group showed MI = 0.354 ± 2.787 vs MII = 3.962 ± 3.584 (mean ± SD; per-phase n = 30). The planned analysis uses a two-sample, two-sided test at α = 0.05; subgroup counts were corrected to 30 per phase (total N = 120 across NOR/DOR × MI/MII). Using the observed SDs as variance estimates for sensitivity (not "observed/post-hoc" power), the pooled SD was 3.210. The observed mean difference was 3.608 ΔCt, corresponding to Cohen's d = 1.124 (Hedges' g = 1.109); the approximate 95% CI for d was 0.58 to 1.67. The difference-in-means had an approximate 95% CI of 1.95 to 5.27 ΔCt. With n = 30 per group, the minimal detectable difference (MDE) at 80% power and α = 0.05 (two-sided) is about 2.32 ΔCt (computed as $\Delta * \approx (z1-\alpha/2 + z1-\beta\ )sp\ \sqrt{2}/n$)). Because the observed difference (3.61 ΔCt) materially exceeds the MDE (2.32 ΔCt), the available sample size provides adequate sensitivity for the primary contrast. We emphasize that we report effect sizes with 95% CIs and MDE, rather than "observed power," because the latter is mathematically redundant with the p-value and adds little inferential value. Assumptions for the sensitivity calculation were equal variances and independent observations; when distributional assumptions are not strictly met, the calculation is conservative. As a result, NOR-stratum CCNA2 (MI vs MII) was chosen for power justification; with n = 30 per phase, pooled SD = 3.210, and α = 0.05, the MDE is ~2.32 ΔCt, while the observed difference is ~3.61 ΔCt (d ≈ 1.12; 95% CI for d: 0.58–1.67), supporting the adequacy of the sample size for this primary comparison

Supplement Materials 2 (S2): The Features of the Samples

Suppl Table S1 The Features of the sample. The RNA and cDNA amounts of samples and the used amount of camples in both cDNA reverse transcription and qRT-PCR

GROUPS (NOR/DOR)	RNA METRICS		GV/MI (IMMATURE)	G2 /(MATURE)	CDNA QUANTITY	RT-PCR QUANTITY
	RNA (ng/µl)	For 200 ng (µl)			cDNA (ng/µl)	For 100 ng (µl)
**3.DOR (MII)**	14,516	13		MII	704,442	0.15
**5.DOR (MI)**	14,968	13,3	MI		868,740	0,23
**5.DOR (MII)**	36,057	5,5		MII	1075,115	0,19
**6.DOR (MI)**	18,03	11,1	MI		1150,070	0,17
**6.DOR (MII)**	14,742	14		MII	1511,4	0,13
**10.DOR (MII)**	25,914	7,7		MII	1190,24	0,17
**11.DOR (MI)**	15,05	13,2	MI		733,68	0,27
**12.DOR (MII)**	15,06	13,2		MII	1230,41	0,16
**13.DOR (MI)**	49,363	4,04	MI		1416,425	0,14
**14.DOR (MII)**	21,287	9,4		MII	1254,755	0,16
**16.DOR (MII)**	17,813	11,2		MII	1885,695	0,11
**17.DOR (MI)**	25,333	7,9	MI		1768,295	0,11
**18.DOR (MI)**	23,581	8,3	MI		1688,315	0,12
**19.DOR (MI)**	13,595	14,2	MI		1603,185	0,12
**19.DOR (MII)**	18	11,2		MII	1611,260	0,12
**21.DOR (MI)**	19,838	10	MI		1287,13	0,16
**21.DOR (MII)**	52,583	3,77		MII	1620,265	0,12
**22.DOR (MI)**	18,501	10,52	MI		920,65	0,22
**22.DOR (MII)**	15,355	13,3		MII	868,9	0,23
**23.DOR (MI)**	11,915	16,67	MI		1238,56	0,16
**24.DOR (MII)**	14,292	14,2		MII	1199,725	0,17
**26.DOR (MI)**	25,224	8	MI		989,695	0,20

**26.DOR (MII)**	25,325	8	MI	MII	1303,95	0,15
**27.DOR (MI)**	23,255	8,69	MI		1371,715	0,15
**28.DOR (MII)**	33,604	5,88		MII	1411,095	0,14
**29.DOR (MII)**	66,681	2,98		MII	1322,795	0,15
**30.DOR (MI)**	34,977	5,71	MI		987,765	0,20
**30.DOR (MII)**	92,638	2,15		MII	1143,905	0,17
**31.DOR (MI)**	31,02	6,45	MI		1315,705	0,15
**31.DOR (MII)**	31,406	6,45		MII	1325,14	0,15
**32.DOR (MII)**	85,75	2,32		MII	1399,37	0,14
**33.DOR (MII)**	41,683	4,76		MII	858,91	0,23
**34.DOR (MII)**	49,114	4,08		MII	1002	0,20
**35.DOR (MII)**	53,927	3,7		MII	1248,945	0,16
**36.DOR (MII)**	25,031	8		MII	1150,16	0,17
**37.DOR (MII)**	70,154	2,84		MII	1229,515	0,16
**38.DOR (MII)**	83,206	2,4		MII	1367,81	0,15
**39.DOR (MII)**	64,572	3,07		MII	1181,48	0,17
**40.DOR (MI)**	99,314	2	MI		1426,05	0,14
**40.DOR (MII)**	118,302	1,7		MII	1322,815	0,15
**41.DOR (MI)**	142,911	1,4	MI		1371,775	0,15
**42.DOR (MI)**	73,077	2,73	MI		1417,925	0,14
**42.DOR (MII)**	62,474	3,21		MII	1230,6	0,16
**43.DOR (MII)**	307,417	0,65		MII	1745,58	0,11
**44.DOR (MI)**	75,587	2,64	MI		819,18	0,24
**44.DOR (MII)**	117,157	1,71		MII	1385,24	0,14
**45.DOR (MI)**	92,23	2,16	MI		1209,445	0,17
**45.DOR (MII)**	105,246	1,9		MII	761,19	0,26
**46.DOR (MII)**	131,839	1,52		MII	855,2	0,23

**47.DOR (MII)**	78	2,56		MII	1008,76	0,20
**48.DOR (MII)**	117,662	1,7		MII	758,885	0,26
**49.DOR (MI)**	42,81	4,67	MI		1064,09	0,19
**49.DOR (MII)**	30,436	6,57		MII	524,4	0,38
**50.DOR (MI)**	85,756	2,33	MI		986,735	0,20
**50.DOR (MII)**	104,497	1,91		MII	710,46	0,28
**51.DOR (MI)**	44,769	4,5	MI		623,62	0,32
**51.DOR (MII)**	80,69	2,,47		MII	304,24	0,66
**52. DOR (MI)**	31,629	6,25	MI		142,965	1,40
**52.DOR (MII)**	32,896	6,06		MII	159,185	1,26
**53.DOR (MII)**	61,926	3,23		MII	356,68	0,56
**54.DOR (MII)**	29,743	6,72		MII	165,215	1,21
**55.DOR (MII)**	40,05	4,99		MII	111,645	1,79
**56.DOR (MII)**	578,694	0,34		MII	1046,74	0,19
**57.DOR (MI)**	630,769	0,317	MI		881,24	0,23
**57.DOR (MII)**	819,446	0,244		MII	1020,125	0,20
**58.DOR (MI)**	374,68	0,534	MI		951,585	0,21
**58.DOR (MII)**	499,004	0,4		MII	875,065	0,23
**59.DOR (MI)**	350,465	0,571	MI		931,805	0,21
**59.DOR (MII)**	392,553	0,509		MII	1559,065	0,13
**60.DOR (MI)**	294,642	0,68	MI		992,985	0,20
**1.NOR (MI)**	16,741	12	MI		1334,419	0,11
**1.NOR (MII)**	17,261	12		MII	1539,485	0,13
**2.NOR (MI)**	63,419	3,15	MI		1041,03	0,19
**2.NOR (MII)**	19,827	10,1		MII	1408,17	0,14
**3.NOR (MI)**	27,646	7,2	MI		1050,425	0,19
**3.NOR (MII)**	41,231	4,9		MII	984,405	0,20

**4.NOR (MII)**	33,534	6		MII	1712,15	0,12
**5.NOR (MII)**	26,933	7,4		MII	1222,225	0,16
**6.NOR (MII)**	57,536	3,5		MII	789,675	0,25
**7.NOR (MII)**	43,171	5		MII	1285,34	0,16
**8.NOR (MII)**	22,87	8,7		MII	618,695	0,32
**11.NOR (MII)**	13,993	14,2		MII	687,6	0,29
**12.NOR (MII)**	27,407	7,4		MII	609,235	0,33
**13.NOR (MII)**	13,595	14,2		MII	607,625	0,33
**14.NOR (MII)**	13,74	14,2		MII	781,98	0,26
**15.NOR (MII)**	16,449	12,5		MII	551,86	0,36
**16.NOR (MII)**	14,674	13,3		MII	1019,845	0,20
**17.NOR (MII)**	34,727	5,71		MII	647,015	0,31
**18.NOR (MII)**	13,428	14,9		MII	1145,365	0,17
**19.NOR (MII)**	69,868	2,85		MII	781,03	0,26
**20.NOR (MII)**	19,815	10		MII	1684,825	0,12
**22.NOR (MII)**	88,35	2,26		MII	1433,92	0,14
**23.NOR (MII)**	35,702	5,6		MII	1191,565	0,17
**24.NOR (MII)**	123,005	1,63		MII	1382,57	0,14
**25.NOR (MII)**	29,833	6,71		MII	1577,32	0,13
**26.NOR (MII)**	43,458	4,6		MII	1150,38	0,17
**27.NOR (MII)**	121,242	1,65		MII	511,575	0,39
**28.NOR (MII)**	52,667	3,8		MII	1402,29	0,14
**29.NOR (MII)**	39,488	5,06		MII	658,155	0,30
**30.NOR (MII)**	44,334	4,51		MII	813,81	0,25
**31.NOR (MII)**	93,617	2,14		MII	731,395	0,27
**32.NOR (MII)**	55,669	3,6		MII	1210,335	0,17
**33.NOR (MI)**	44,973	4,45	MI		729,355	0,27

**34.NOR (MI)**	56,48	3,54	MI		1283,345	0,16
**35.NOR (MI)**	41,748	4,79	MI		762,94	0,26
**36.NOR (MI)**	44,125	4,53	MI		1034,09	0,19
**37.NOR (MI)**	52,97	3,77	MI		912,52	0,22
**38.NOR (MI)**	41,484	4,82	MI		1420,645	0,14
**39.NOR (MI)**	56,573	3,53	MI		1201,605	0,17
**40.NOR (MI)**	93,309	2,14	MI		1279,875	0,16
**41.NOR (MI)**	24,035	8,32	MI		1696,460	0,12
**42.NOR (MI)**	52,651	3,8	MI		1017,885	0,20
**43.NOR (MI)**	470,366	0,42	MI		903,285	0,22
**44.NOR (MI)**	392,62	0,51	MI		729,725	0,27
**45.NOR (MI)**	318,029	0,62	MI		726,285	0,28
**46.NOR (MI)**	244,823	0,81	MI		675,86	0,30
**47.NOR (MI)**	970,599	0,2	MI		675,345	0,30
**48.NOR (MI)**	260,791	0,76	MI		514,565	0,39
**49.NOR (MI)**	196,781	1,02	MI		952,02	0,21
**50.NOR (MI)**	404,601	0,49	MI		1313,81	0,15
**51.NOR (MI)**	297,375	0,67	MI		935,59	0,21
**52.NOR (MI)**	43,793	4,5	MI		1064,555	0,19
**53.NOR (MI)**	42,794	4,6	MI		1395,46	0,14
**54.NOR (MI)**	25,099	8	MI		1533,765	0,13
**55.NOR (MI)**	34,287	5,88	MI		1230,01	0,16
**56.NOR (MI)**	72,617	2,74	MI		853,99	0,23
**57.NOR (MI)**	32,714	6,06	MI		1208,68	0,17
**58.NOR (MI)**	1171,458	0,17	MI		1174,78	0,17
**59.NOR (MI)**	22,194	9,09	MI		1210,695	0,17
**60.NOR (MI)**	46,321	4,34	MI		1321,001	0,15

**61.NOR (MI)(BACKUP)**	44,897	4,44	MI		1332,345	0,15

***NOR: Normoresponder; **DOR: Diminished Over Reserve; ***MI: Mitophase I; ****MII: Mitophase II**

Suplementary Materials S3: Stratified Diagnostic-Performance Analysis

Specifically, we fit multivariable logistic models to distinguish NOR vs DOR within each oocyte phase (MI and MII), using CCNA2, CCNB3, miR-17, miR-106b, miR-1275 with age and BMI as prespecified covariates. Discrimination was summarized by AUC with 95% CIs, and threshold metrics were reported at the Youden-J cut-point.

• MI subset (NOR vs DOR): AUC 0.863 (SE 0.056; 95% CI 0.753–0.974; BCa 95% CI 0.723–0.950; p<0.0001). At the Youden-J threshold ($\hat{p}$ >0.666), sensitivity 72.4% and specificity 100%. This indicates good discrimination with perfect rule-in at the optimal cut-point.

• MII subset (NOR vs DOR): AUC 0.971 (SE 0.020; 95% CI 0.933–1.000; BCa 95% CI 0.898–0.996; p<0.0001). At the Youden-J threshold ($\hat{p}$ ≈0.539), sensitivity 93.1% and specificity 96.6%, indicating excellent and well-balanced accuracy.

<u>ROC Figures</u>

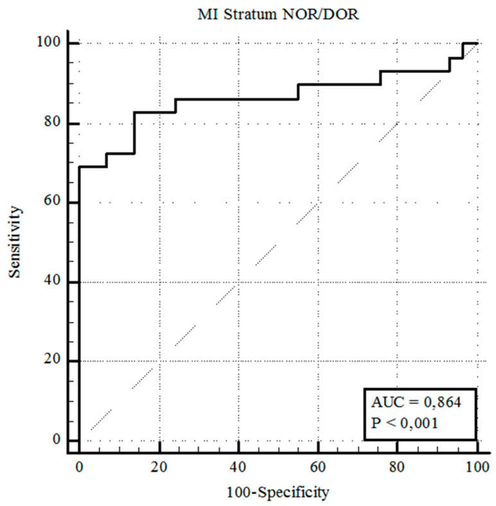

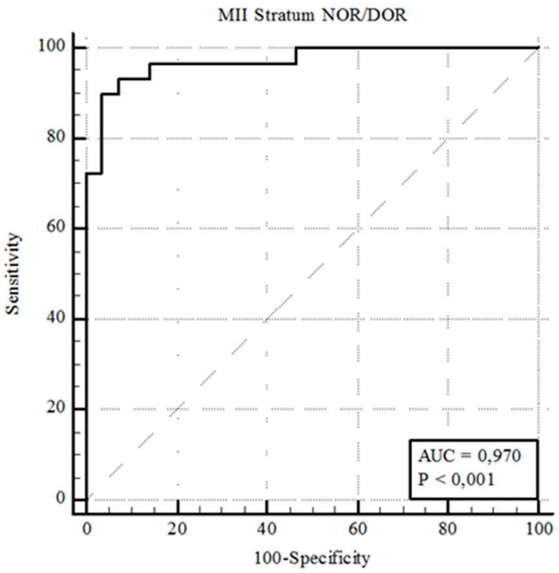
MII Stratum NOR/DOR
Sensitivity
100-Specificity
0
20
40
60
80
100
AUC = 0,970
P < 0,001
